# Synergistic Effects of Silicon and Aspartic Acid on the Alleviation of Salt Stress in Celery (*Apium graveliens* L.) “Si Ji Xiao Xiang Qin”

**DOI:** 10.3390/plants13152072

**Published:** 2024-07-26

**Authors:** Jinnan Song, Jingli Yang, Byoung Ryong Jeong

**Affiliations:** 1 Shandong Provincial University Laboratory for Protected Horticulture, Weifang University of Science and Technology, Shouguang 262700, China; yangmiaomiaode@gmail.com; 2 Division of Horticultural Science, College of Agriculture and Life Sciences, Gyeongsang National University, Jinju 52828, Republic of Korea

**Keywords:** plant biomass, photosynthesis, ion uptake, nutritious status, antioxidants

## Abstract

Salinity is one of the primary abiotic stresses that seriously hampers plant quality and productivity. It is feasible to reduce or reverse the negative effects of salt through the supplementation of silicon (Si) and aspartic acid (Asp). However, the question of how exogenous Si and Asp induce salt tolerance in celery remains incipient. Thus, this study was performed to determine the synergistic effects of Si and Asp on the alleviation of salt stress in celery. To this end, the celery plants were cultivated in a controlled regime (light for 14 h at 22 °C; darkness for 10 h at 16 °C) and treated with one of five treatments (CK, 100 mM NaCl, 100 mM NaCl + 75 mg/L Si, 100 mM NaCl + 100 mg/L Asp, and 100 mM NaCl + 75 mg/L Si + 100 mg/L Asp). Results showed that solely NaCl-treated celery plants developed salt toxicity, as characterized by decreased growth, declined photosynthetic ability, disturbed nutritious status and internal ion balance, and a boosted antioxidant defense system (Improved antioxidant enzymes and reduced ROS accumulation). In contrast, these adverse effects of NaCl were ameliorated by the additions of Si and Asp, regardless of Si, Asp, or both. Moreover, the mitigatory impacts of the co-application of Si and Asp on salt stress were more pronounced compared to when one of them was solely applied. Collectively, exogenous Si and Asp alleviate the degree of salt stress and thereby improve the salt tolerance of celery.

## 1. Introduction

Agricultural sustainability and productivity are being threatened by multiple negative influences on crops, such as climate change [1], together with plant homeostatic instability by global warming and water and nutrient limitation [2,3]. Moreover, indisposed irrigations are increasing soil salinity levels; salinity is one of the predominant harmful abiotic factors that restrict a plant’s growth, quality, and yield worldwide [4]. Intuitively, salinity soils account for 20% of the worldwide agricultural land, and approximately 800 million hectares of soil globally are being highly affected by a high salt content [5,6]. Soil salinization is considered a major cause of land degradation in both arid and semiarid regions [7].

Meanwhile, salt stress could disturb a plant’s growth and development in all stages [8]. According to previous reports, the most common adverse effects of salinity on plants are an interrupted reactive oxygen species (ROS) detoxification system, thereby causing impaired redox homeostasis, membrane damage, plasmolysis, and even nutritional disturbances and toxicity [6,9,10,11]. Physiologically, the plant’s growth regarding its roots, shoots, and leaves were notably inhibited [6,12]. Morphologically, the plants were subjected to being stunted [8,13]. Metabolically, the cellular homeostasis regarding multiple biomolecules, such as lipids, chlorophylls, proteins, and nutrition ions, was severely damaged [8,13,14,15].

Higher plants have developed sophisticated mechanisms to cope with the toxic influences of salt stresses. For instance, plants will activate the ROS scavenge mechanism by enhancing the antioxidant defense system for a reduction in ROS [16]. Usually, the synthesis and accumulation of osmoprotectants or suitable osmolytes are common mechanisms where plants can overcome abiotic stresses [17,18]. A plant’s antioxidant defense system is composed of a nonenzymatic antioxidant system and an antioxidative enzymatic system [19], such as the antioxidant enzymes regarding superoxide dismutase (SOD), peroxidase (POD), and catalase (CAT) [20].

Celery (*Apium graveliens* L.). is an annual or perennial old herb belonging to the *Apiaceae* family and is widely distributed nationwide [21]. As a salad vegetable, it has been ranked second in global consumption due to the following reasons [22]: Environmentally, celery was adopted in a drainage solution reuse system for the reduction in pollutants by wastewater and excrescent chemicals [23]. Medicinally, celery can prevent liver and lien diseases, jaundice, and cardiovascular diseases due to the fact that it contains abundant nutrients regarding the apigenin coumarins, vitamins, carotene, volatile oil, flavonoids, etc. [24,25,26]. Most of the previous studies on celery have focused on its therapeutic roles [27], celery food chemistry [28], cultivation regime [29], and abiotic stress effects on celery development [21,30,31,32], neglecting the effective approach to the alleviation of stresses, in particular of salt stress. And the yield and quality of celery were severely restricted by salt stress.

To reduce the salt damages on the plant quality and production, attempts have been made to mitigate soil salinization and improve plant salt tolerances [33,34]. For instance, researchers implemented leaching and flushing to reduce the salt in the soil, which is time-consuming and labor-intensive [35]. Phytoremediation is another way, and it has been shown to be eco-friendly but not effective [33,35]. Moreover, applying exogenous substances, such as silicon (Si) [36] and amino acid (AA) [32], is a method to promote the salt tolerance of plants. And this approach, which is associated with agronomical means, remains the most reliable avenue for minimizing the adverse impacts of salinity.

Silicon (Si) is one of the most abundant elements in the Earth’s crust and was recently considered a “quasi-essential” element, according to the International Plant Nutrition Institute [37]. Although Si is not listed as an essential element, supplementation of Si has been suggested as beneficial for plant growth regarding multiple aspects, such as improved yield ability and enhanced resistance against disease and abiotic stresses [38]. More specifically, Si is often used as an attenuator to reduce the adverse impacts of salinity, and moreover, its promotion of a plant’s salt tolerance could be ascribed to many physiological improvements. The amorphous Si could be deposited on the leaf epidermis, regulating the stomatal conductance and transpiration rate, which favors photosynthesis [39]. Si participates in the rigidity of cell walls, constituting a better leaf architecture and leaf area and greater light interception, which also increases the net photosynthetic rate [39,40]. Meanwhile, Si has been shown to interact with certain cations, such as Na^+^ and K^+^; thus, the important role of Si in the alleviation of salt toxicity is the reduction in Na^+^ accumulation and facilitating Na^+^ exclusion [38]. In addition, previous reports disclosed that the application of Si can decrease lipid peroxidation and maintain redox homeostasis by reinforcing the antioxidant defense system [40,41]. Therefore, Si is believed to be an effective method to improve the salt tolerance and quality of plants.

Aspartic acid (Asp), also known as Aspartate, is a basic constituent of proteins and plays an important role in metabolic energy production and equivalent reductions [42,43]. Asp participates in the plant metabolism pathway for the synthesis of important molecules, such as organic acids, nucleotides, and hormones [43]. Meanwhile, considerable reports have shown that Asp is required in the regulation process when facing adverse conditions, particularly regarding salinity [42,44]. In high salt environments, Asp has been found to accumulate with proline in osmotic adjustments and membrane stability on the basis of the physiological responses, even though the underlying metabolic mechanism remains unclear [18,44,45]. Thus, these beneficial influences on plant growth and development conferred by exogenous Asp have encouraged more researchers to apply Asp to other plants, especially for salt-sensitive crops.

However, the synergistic effects of Si and Asp on the alleviation of salt stress in celery have rarely been reported. Therefore, the main objective of the study undertaken herein on celery is to (1) investigate whether exogenous Si or Asp is able to reduce the salt stress degree in celery; concomitantly, to (2) assess the combined effects of Si and Asp on the physiology, morphology, photosynthesis, nutrition status, and antioxidant defense system in salt-stressed celery.

## 2. Materials and Methods

### 2.1. Plant Material and Growing Conditions

The celery seeds “Si Ji Xiao Xiang Qin” were purchased from LuTong Seed Company., Ltd. (Handan, China) with a mean germination ratio of over 70%. The celery seeds were planted in 128-cell plug trays containing a mini-K substrate (Klasmann-Deilmann GmbH Company, Geeste, Germany) and moistened with running tap water. The celery seeds germinated 7 days after sowing (DAS) in an air-conditioned environment at 20 °C and a relative humidity of 80%. Then, the germinated seedlings were watered with MNS (multiple nutrition solutions, pH = 6.0), according to our previous reports [46]. The seedlings were cultured in a controlled alternating diurnal regime with 14 h light (white LED at 800 µmol m^−2^ s^−1^ PPFD) and 10 h dark, at 22 °C and 16 °C, respectively, and the relative humidity was 70% [32]. The celery seedlings were allowed to grow for another 7 days (14 DAS) until they turned into two true leaves and one heart. The seedlings with uniform size and similar morphology but without mechanical flaws were monitored, selected, and transferred to new 128-cell plug trays.

### 2.2. Treatments and Experimental Design

Subsequently, the transferred celery seedlings were equally divided into 5 parts and treated with the following 5 combinations: CK (0 mM NaCl + 0 mg/L Si + 0 mg/L Asp), NaCl (100 mM NaCl + 0 mg/L Si + 0 mg/L Asp), NaCl + Si (100 mM NaCl + 75 mg/L Si + 0 mg/L Asp), NaCl + Asp (100 mM NaCl + 0 mg/L Si + 100 mg/L Asp), and NaCl + Si + Asp (100 mM NaCl + 75 mg/L Si + 100 mg/L Asp). NaCl was directly dissolved in the MNS, and Si was sourced from K_2_SiO_3_; thus, the excessive introduced potassium was reduced by KNO_3_, and the resultant losses of nitrate were balanced by nitric acid [46]. The optimized level of K_2_SiO_3_ was at 75 mg/L, following our previous finding [47]. All the plants were watered with the treatment solutions only, and all the treatment solutions were irrigated every alternative day until harvest. The Asp solution at 100 mg/L was foliar sprayed twice with a 7-day interval (on 15 DAS and 22 DAS) [18].

This experiment is laid out in a completely randomized design with three biological replications. For each replicate, 16 celery seedlings underpinning one treatment were adopted.

### 2.3. Measurement of Growth Parameters and Destructive Sampling

The celery plants were harvested until they showed distinct appearances (39 DAS). During the harvest, the plant growth parameters from different treatments were individually determined. The whole plant weights in terms of fresh weight and whole dry mass (kept in an air-force oven at 60 °C for 72 h) were determined by an electronic balance. The stem diameter was measured using a Vernier caliper (SJ-455520, ShangJiang Instrument Co., Ltd., Haining, China). The shoot length, leaf length and width, and tap root length were recorded with a metal ruler. The celery plants from different treatments were individually sampled, immediately frozen in liquid N_2_, and stored in a refrigerator at −80 °C until further experiments.

### 2.4. Estimation of Net Photosynthesis, Transpiration Rates, Stomatal Conductance, and Chlorophylls

The net photosynthesis (*P*n), transpiration rates (*T*r), and stomatal conductance (*g*_s_) were determined with a hand-held photosynthesis measurement system (TARGAS-1, PP Systems, Amesbury, MA, USA). Briefly, these parameters were measured on the three topmost fully expanded leaves, and the measurement per leaf was conducted three times. The leaf temperature was about 23 °C, and the environment during measuring was identical with that previously set when growing celery.

The chlorophyll contents (chlorophyll a and b) were measured following Arnon’s reports with minor modifications [48]. Briefly, 0.1 g of fresh celery leaves was mixed with a 2 mL extraction buffer (45% *v*/*v* acetone, 45% *v*/*v* ethanol, and 10% *v*/*v* H_2_O) and kept at 4 °C overnight. A mild shaking was carried out with a rotator (AG, FINEPCR, Seoul, Republic of Korea) during the incubation. After the incubation, the supernatant was transferred and spectrophotometrically read at 645 nm, 663 nm, and 440 nm with a spectrophotometer (UV3200, OptoSky, Xiamen, China). Afterwards, the contents of chlorophyll a and chlorophyll b, together with the carotenoids, were individually calculated with the following formulae:

$$\mathrm{Chlorophyll}\;a=\frac{\left(12.72\;\times \;{\mathrm{OD}}_{663}-\;2.59\;\times \;{\mathrm{OD}}_{645}\right)\;\times \;V}{\mathrm{Sample}\;\mathrm{fresh}\;\mathrm{weight}}$$

$$\mathrm{Chlorophyll}\;b=\frac{\left(22.88\;\times \;{\mathrm{OD}}_{645}-\;4.67\;\times \;{\mathrm{OD}}_{663}\right)\;\times \;V}{\mathrm{Sample}\;\mathrm{fresh}\;\mathrm{weight}}$$

$$\mathrm{Carotenoids}=\frac{\left.4.7\;\times \;{\mathrm{OD}}_{440}-\;0.27\;\times \;(\mathrm{Chl}\;a+\mathrm{Chl}\;b\right)}{\mathrm{Sample}\;\mathrm{fresh}\;\mathrm{weight}}$$

where ‘V’ refers to the volume used of the extraction buffer (‘V’ was 2 mL herein), and the chlorophyll content is quantified by milligrams per gram of fresh-weight leaves.

### 2.5. Determination of Soluble Sugar, Starch, and Soluble Protein

The soluble sugar and starch contents were determined following an anthrone–sulfuric acid colorimetry approach according to McCready’s reports with minor modifications [49].

Briefly, 0.5 g of finely ground celery leaf powder was vigorously mixed with 25 mL deionized water and incubated in a 90 °C water bath for 40 min. Then, the mixture was subjected to centrifugation (6500 rpm, 10 min, RT) to collect the supernatant. A total of 0.1 mL of the supernatant was mixed with distilled water and 2% (*w*/*v*) anthrone (dissolved in ethyl acetate) at 1.9 mL and 1 mL, respectively. A total of 5 mL concentrated H_2_SO_4_ was slowly added to the mixture and was subjected to a 90 °C water bath for 10 min. Finally, the absorbance was recorded at 630 nm, and the soluble sugar content was calculated on the basis of a standard soluble sugar curve. The residue after centrifugation from the previous steps was collected for the determination of starch; the detailed procedure can be found in our previous publication [50].

The soluble protein contents were determined with a Bradford reagent [51].

### 2.6. Quantifications of Na, K, Ca, and Mg

The celery leaf samples from each treatment were harvested and washed with distilled water to remove foreign particles. Then, they were placed in an oven at 70 °C until they had a constant weight. All the samples were finely ground into powder and wet-ashed to break all the organic matrix, leaving only the minerals for analysis. The ion contents (Na, K, Ca, and Mg) in the digested transparent solution were determined with Atomic Absorption Spectrophotometry. The detailed digesting method, decoction method, and calibration procedure can be found in Sahito’s report [52].

### 2.7. Determination of Antioxidant Enzyme Activities and ROS (O_2_^·−^, H_2_O_2_) Contents

The antioxidant enzyme activities, herein consisting of SOD, POD, CAT, and APX, were calculated on the basis of the determined soluble protein contents mentioned above. The SOD concentration was determined according to the nitro blue tetrazolium (NBT) inhibition method [53]. POD activity was determined by adopting the guaiacol oxidation reaction [54]. CAT activity was measured based on the H_2_O_2_ decomposition [55]. APX activity was determined on the basis of the H_2_O_2_ scavenging degree [56]. The mentioned antioxidant enzyme activities were all spectrophotometrically determined with a spectrophotometer (UV3200, OptoSky, Xiamen, China).

A principle of hydroxylamine oxidization was used for measuring the superoxide (O_2_^·−^) level according to a protocol by Wu [57]. The Hydrogen Peroxide (H_2_O_2_) content was determined following a rapid and sensitive approach, as presented by Uchida [58].

### 2.8. Statistics and Graphing

All the displayed data in this experiment are the means ± SE of no less than three biological replicates (*n* ≥ 3). The obtained data were subjected to a one-way ANOVA following Duncan’s multiple comparison range test at *p* = 0.05 with SAS statistical software 8.2, and the significant differences are shown by different lowercase letters over bars. The bar graphs were created with GraphPad Prism 8.2 software. The correlation heat map was plotted in an Origin 2022 procedure.

## 3. Results

### 3.1. The Celery Growth Parameters as Affected by NaCl, Si, and Asp

**Figure 1:**
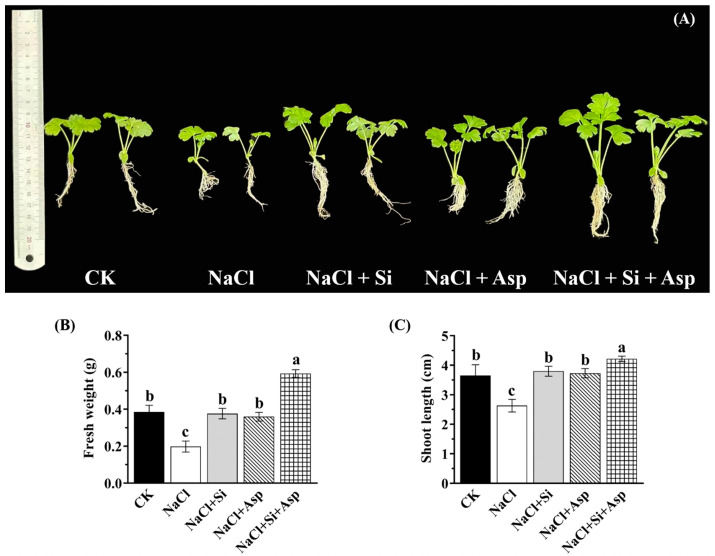
Effect of exogenous Si and Asp on the (**A**) morphology and growth parameters regarding (**B**) fresh weight and (**C**) shoot length of celery plants under salt stress. Data are means ± SE generated from *n* = 6 biological replicates. The significant differences among treatments were determined according to Duncan’s multiple comparison range test when *p* = 0.05 (One-way ANOVA) and shown by different lowercase letters over bars.

The celery plants showed distinct changes in response to the salt stress, exogenous Si, and Asp treatments (Figure 1). The growth and morphology were notably inhibited when treated with NaCl compared to CK. However, the supplementation of Si or Asp significantly promoted the growth compared to CK. Solely Si- or Asp-treated celery plants displayed similar morphology to CK, while the co-application of Si and Asp dramatically increased the growth and morphology.

In a large number of celery plants, the fresh weight and shoot length could directly reflect the plant’s growth ability. As depicted in Figure 1B,C, salt treatment significantly decreased the fresh weight and shoot length by 38.8% and 27.9%, respectively, when compared with CK (Figure 1B,C). However, the supplementation of Si or Asp alone markedly improved the fresh weight by 90.4% and 81.8%, respectively, while the co-application of Si and Asp significantly improved this parameter by 1.98-fold when compared with that cultured with NaCl (Figure 1B). Similarly, the shoot length in the ‘NaCl + Si + Asp’ regime significantly improved by 60.5% relative to that treated with NaCl (Figure 1C).

### 3.2. Other Main Growth Parameters as Affected by NaCl, Si, and Asp

**Figure 2:**
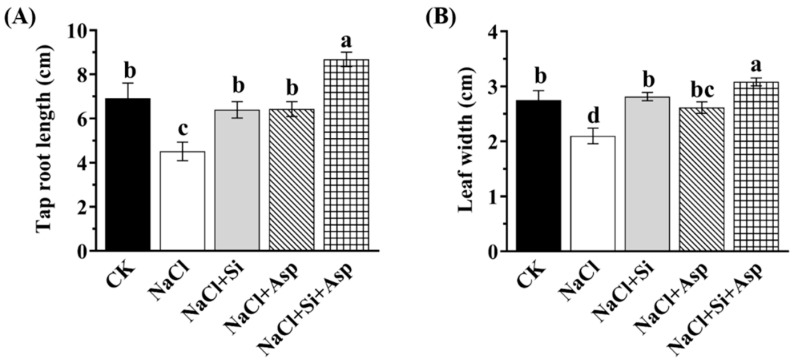
Effect of exogenous Si and Asp on the (**A**) tap root length and (**B**) leaf width of celery plants under salt stress. Data are means ± SE generated from *n* = 6 biological replicates. The significant differences among treatments were determined according to Duncan’s multiple comparison range test when *p* = 0.05 (One-way ANOVA) and shown by different lowercase letters over bars.

Consistent with the growth status depicted in Figure 1, other major investigated parameters regarding the tap root length, leaf length and width, whole dry weight, and stem diameter were also determined. These parameters were all reduced in response to the NaCl treatment, while no significant differences were conferred among CK, NaCl + Si, and NaCl + Asp (Figure 2; Table 1).

However, conspicuously, compared with the celery plants treated with NaCl, the supplementation of either Si or Asp or co-application of both substances significantly improved these determined parameters (Figure 2; Table 1). As the most important finding, the collegial use of Si and Asp further dramatically increased these parameters compared with that treated with Si or Asp alone (Figure 2; Table 1).

### 3.3. The Photosynthetic Responses to the NaCl, Si, and Asp

**Figure 3:**
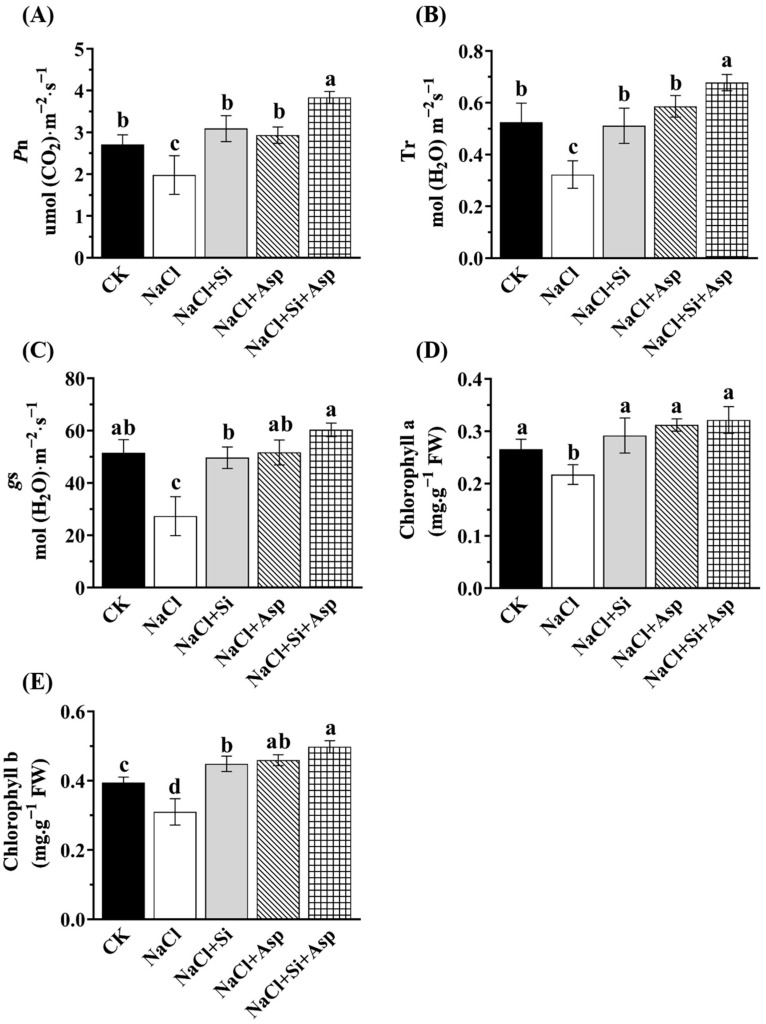
Effect of exogenous Si and Asp on the photosynthetic capacity regarding (**A**) the net photosynthesis (*P*n), (**B**) transpiration rates (*T*r), (**C**) the stomatal conductance (*g*_s_), (**D**) chlorophyll a content, and (**E**) chlorophyll b content of celery plants under salt stress. Data are means ± SE generated from *n* = 6 biological replicates. The significant differences among treatments were determined according to Duncan’s multiple comparison range test when *p* = 0.05 (One-way ANOVA) and shown by different lowercase letters over bars.

The photosynthetic ability in terms of certain major parameters, such as the net photosynthesis (*P*n), transpiration rates (*T*r), and stomatal conductance (*g*_s_), was determined herein when the celery was cultured in different regimes. We found that all the recorded traits were significantly decreased in response to NaCl treatment (Figure 3). Regarding the more affected parameters, the net photosynthesis rate and the transpiration rates of NaCl-spiked celery plants were significantly decreased by 27.2% and 39.6%, respectively, compared with CK (Figure 3A,B). However, both Si and Asp remarkedly ameliorated the photosynthetic ability, regardless of the determined specific parameters. For instance, the supplementation of Si and Asp significantly improved the stomatal conductance (*g*_s_) by 81.74% and 89.06%, respectively, in comparison with that in the salt-stressed celery (Figure 3C). Similar trends could be found in the modulations of chlorophyll a and b (Figure 3D,E).

### 3.4. Contents of Soluble Sugar, Starch, Soluble Protein, and Carotenoids as Affected by NaCl, Si, and Asp

**Figure 4:**
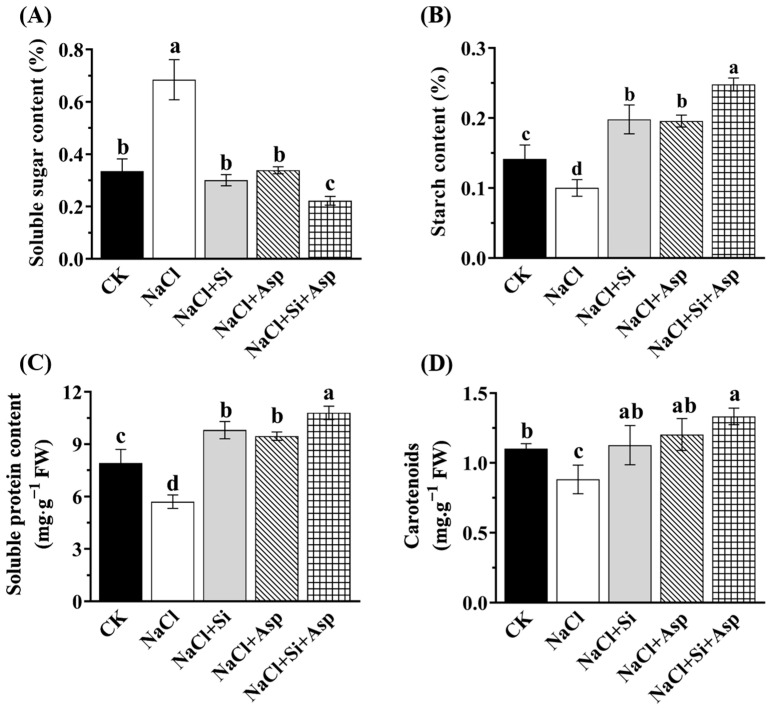
Effect of exogenous Si and Asp on the nutrition parameters regarding (**A**) soluble sugar content, (**B**) starch content, (**C**) soluble protein content, and (**D**) carotenoids content of celery plants under salt stress. Data are means ± SE generated from *n* = 6 biological replicates. The significant differences among treatments were determined according to Duncan’s multiple comparison range test when *p* = 0.05 (One-way ANOVA) and shown by different lowercase letters over bars.

Certain celery internal parameters, such as the soluble sugar, starch, soluble protein, and carotenoids, were determined to reflect the plant’s nutritional status herein (Figure 4). As compared with the CK, the salt-treated celery plants exhibited significant diminishments of the starch content, soluble protein content, and carotenoids. Notably, the soluble sugar content in salt-spiked plants displayed a significant improvement of 104% compared with that in CK (Figure 4A). In contrast, the starch content, soluble protein content, and carotenoids markedly decreased by 28.6%, 28.1%, and 27.3%, respectively (Figure 4B–D).

However, irrespective of the spraying exogenous substances, both Si and Asp significantly upsurged the level of starch, soluble protein, and carotenoids compared with that cultured in the NaCl regime (Figure 4B–D). Moreover, compared with the salt-stressed celery plants, the co-application of Si and Asp considerably improved the starch content, soluble protein content, and carotenoids by 1.5-fold, 89.0%, and 51.1%, respectively (Figure 4). However, we noticed that both Si and Asp significantly declined the soluble sugar content compared to the salt-stressed plants (Figure 4A). In addition, we found that the co-application of Si and Asp significantly increased the starch content and soluble protein content more than Si or Asp used alone (Figure 4B,C).

### 3.5. Na, K, Ca, and Mg Concentration as Affected by NaCl, Si, and Asp

In order to figure out the modulation of internal ion homeostasis when the celery plants were under salt stress and concomitantly determine the correlations among the main ions subjected to five treatments, we further investigated the Na, K, Ca, and Mg contents and assessed the correlations among them.

**Figure 5:**
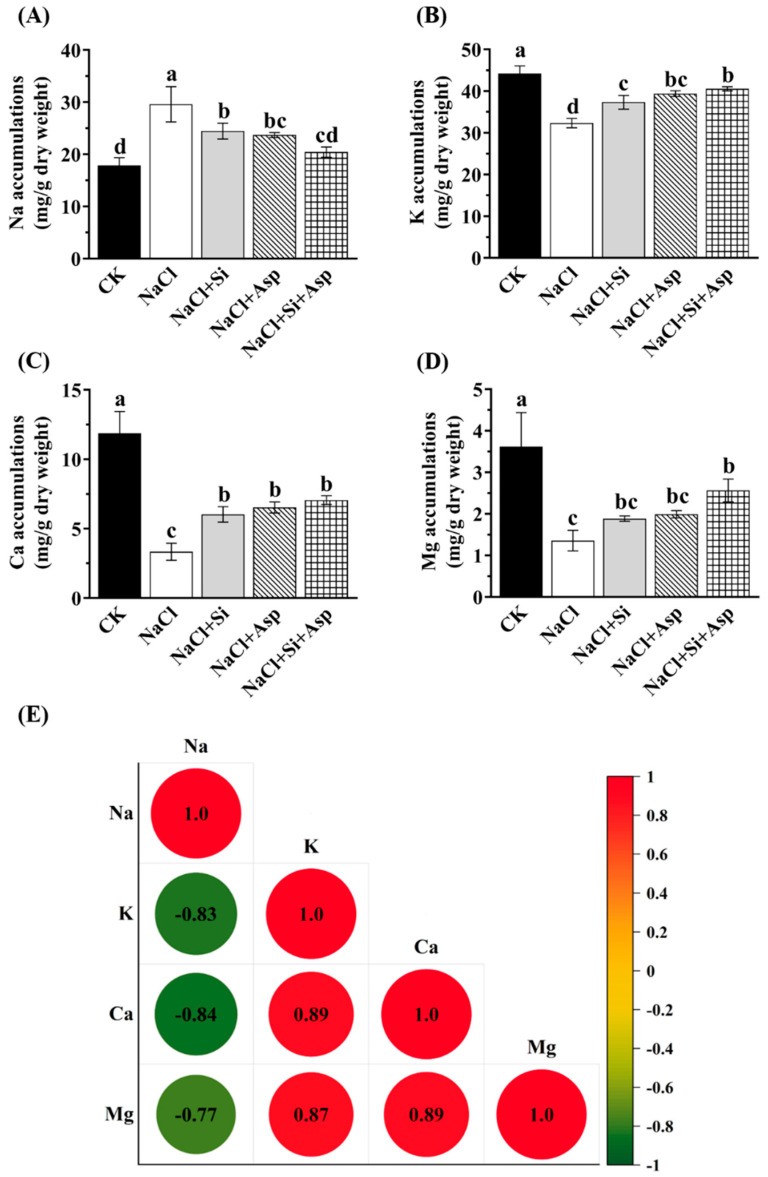
Effect of exogenous Si and Asp on the ion concentration regarding (**A**) Na, (**B**) K, (**C**) Ca, and (**D**) Mg of celery plants under salt stress. Multivariate data analysis of a (**E**) heatmap showing the correlations among these studied ions. Data are means ± SE generated from *n* = 3 biological replicates. The significant differences among treatments were determined according to Duncan’s multiple comparison range test when *p* = 0.05 (One-way ANOVA) and shown by different lowercase letters over bars.

As is apparent in Figure 5, the salt-spiked celery plants exhibited a higher Na content while decreasing the concentrations of K, Ca, and Mg. In other words, the Na in celery plants grew in the NaCl regime significantly by 65.8% compared with CK (Figure 5A); however, the K, Ca, and Mg in that regime significantly decreased by 26.8%, 71.8%, and 62.4%, respectively (Figure 5B–D). By contrast, the added Si and Asp remarkably diminished the Na content while improving the internal level of K, Ca, and Mg. In particular, regarding the co-applications of Si and Asp, the Na level in the ‘NaCl + Si + Asp’ group was 31.01% lower relative to that solely treated with NaCl (Figure 5A).

Consistently, we noticed that the Na content was negatively correlated with K, Ca, and Mg. On the contrary, positive relations among K, Ca, and Mg were monitored (Figure 5E).

### 3.6. Responses of Antioxidant Enzymes Activities to NaCl, Si, and Asp

The oxidative protective system was triggered when the celery plants suffered from salt stress. During this process, the antioxidant capacity, such as the antioxidant enzymes, was improved to reduce the degree of stress. We, therefore, investigated the activities of the main defense antioxidant enzymes regarding SOD, CAT, POD, and APX.

**Figure 6:**
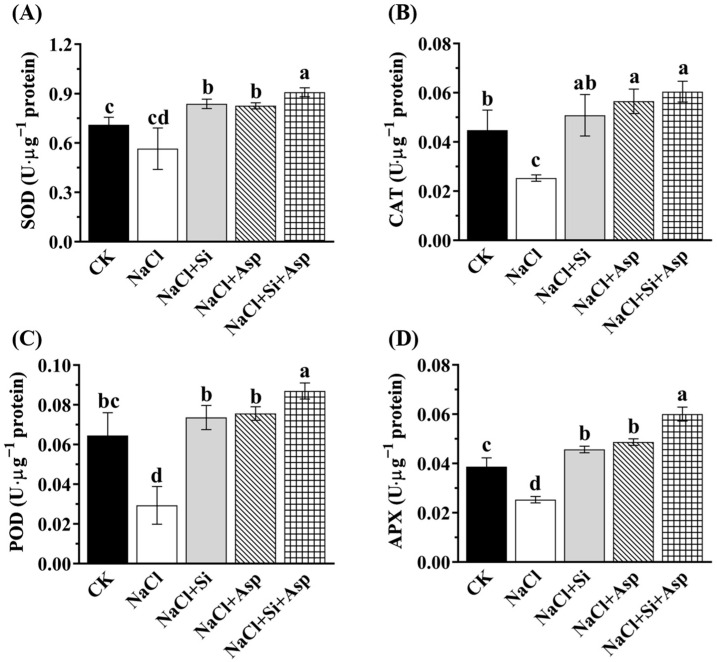
Effect of exogenous Si and Asp on the concentrations of antioxidant enzymes regarding (**A**) SOD, (**B**) CAT, (**C**) POD, and (**D**) APX of celery plants under salt stress. Data displayed are means ± SE generated from *n* = 6 biological replicates. The significant differences among treatments were determined according to Duncan’s multiple comparison range test when *p* = 0.05 (One-way ANOVA) and shown by different lowercase letters over bars.

As compared with CK, the salt-stressed celery plants significantly decreased the CAT, POD, and APX by 44.4%, 1.24-fold, and 56%, respectively (Figure 6B–D). Irrespective of Si or Asp, both of them notably improved these four antioxidant enzymes when compared with those in the salt-spiked celery plants. The co-application of Si and Asp remarkably enhanced SOD, CAT, POD, and APX by 62.5%, 1-fold, 1.97-fold, and 1.4-fold, respectively (Figure 6). In addition, the co-application of Si and Asp significantly improved SOD activity by 10.2% and 8.6%, respectively, than when Si and Asp were used alone (Figure 6A).

### 3.7. Oxidative Damage as Affected by NaCl, Si, and Asp

The oxidative damage could further reflect the antioxidant ability of celery plants, while the former, herein consisting of O_2_^·−^ and H_2_O_2_ levels, were determined.

**Figure 7:**
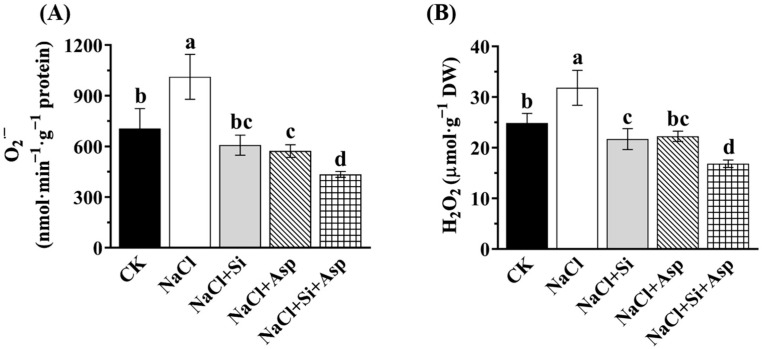
Effect of exogenous Si and Asp on the ROS contents regarding (**A**) O_2_^·−^ and (**B**) H_2_O_2_ of celery plants under salt stress. Data displayed are means ± SE generated from *n* = 6 biological replicates. The significant differences among treatments were determined according to Duncan’s multiple comparison range test when *p* = 0.05 (One-way ANOVA) and shown by different lowercase letters over bars.

Consistent with the above, the celery plants treated with NaCl rapidly increased the accumulations of O_2_^·−^ and H_2_O_2_ by 43.3% and 28.01%, respectively, compared with CK (Figure 7). As expected, both O_2_^·−^ and H_2_O_2_ were significantly diminished after the supplementation of Si and Asp. For instance, the added Si and Asp to the salt-treated celery plants rapidly reduced the O_2_^·−^ content by 31.84% and 31.11%, respectively (Figure 7A).

More importantly, the co-application of Si and Asp dramatically decreased the production of ROS compared with when one of them was solely applied (Figure 7). The H_2_O_2_ content in the ‘NaCl + Si + Asp’ group was significantly lower by 22.34% and 24.26%, compared with that in ‘NaCl + Si’ and ‘NaCl + Asp’, respectively (Figure 7B).

## 4. Discussion

A high salt-spiked supply could inevitably instigate the uptake of Na^+^ by plants, resulting in the imbalance of ions and the inhibition of the plant’s growth ability. This was believed to be the primary cause of salt toxicity [59]. In fact, Si- and Asp-induced alleviations on salt stress have been extensively studied in many plant species, such as pepper [60], rice [41], and wheat [18]. Moreover, Gao recently presented the effects of salt stress on celery and successfully reduced the degree of salt toxicity by using the other amino acid proline [32]. However, the influence of the co-application of Si and Asp on salt-stressed celery has been less understood. Thus, we aimed to confirm the alleviatory effects of Si and Asp on salt-stressed celery plants and concomitantly unveil the associated mechanisms that are responsible for it.

Plant growth and morphology can be adversely affected by high salt content, which has been shown in many plant species, oscillating from crops to vegetables [10,11,12,18]. We also confirmed that the normal growth ability of celery plants was severely disturbed by NaCl (100 mM) treatment (Figure 1 and Figure 2), which is in agreement with Gao’s report [32]. However, this adverse effect was notably mitigated by Si and Asp in terms of the morphology and growth parameters (Figure 1 and Figure 2). Several reports have demonstrated that the application of Si could remarkably reduce the degree of salt toxicity. In addition, the supplementation of Si considerably promoted plant growth and development [41,46,60,61]. Indeed, using Si was solely found to alleviate the salt-stressed degree in celery, as evidenced by the ameliorated whole biomass and multiple main growth traits (Figure 1 and Figure 2). It is worth noting that the water content of plants significantly influenced the uptake and accumulation of Si, showing quadratic behavior [36,38]. However, this effect is minor for the obtained outcome due to the identical water scheme and irrigation frequency. These findings were not only in corroboration with the report by Rohanipoor but also suggest the beneficial effects conferred by Si, in particular for the plants at risk of salt stress [62]. Likewise, the elevation of salt tolerance by the addition of Asp has been found in wheat [18], tomato [11], and onion [42]. And the results shown by this study are in line with these earlier reports: the growth of salt-stressed celery notably increased after the addition of Asp (Figure 1 and Figure 2). Moreover, the growth parameters of salt-stressed celery plants were more promoted when Si and Asp were simultaneously applied than when one of them was solely used, illustrating the synergistic effects between them.

Photosynthetic performance was regarded as one of the most important processes modulating the overall yield ability of plants [63]. Salt toxicity would firstly over-produce the ROS, resulting in oxidative damage and eventually the membrane’s instability, together with the decline of its photosynthetic capacity [41,63,64]. Indeed, our study showed that the investigated photosynthesis-related parameters of salt-stressed celery plants were significantly decreased compared with CK (Figure 3). The net photosynthetic rate (*P*n) is correlated with plant growth and carbohydrate demand, which could directly refer to the accumulation of organic matter [65]. Similarly, as a basic physiological activity, transpiration is associated with heat transfer and directly determines the photosynthesis and yield of crops [66]. In addition, Sakoda noted that a high photosynthetic rate is usually accompanied by a high stomatal conductance (positive correlation) [67]. This phenomenon could probably be attributed to the finding that plants with more stomata tend to have a higher net CO_2_ assimilation rate [67,68]. Meanwhile, photosynthesis is also an intricate mechanism pertaining to the synthesis of photosynthetic pigments. Some studies underpinning various plant species disclosed that the chlorophyll contents were positively correlated with the photosynthetic ability [40,46,69,70]. Consistently, these parameters were significantly improved after the addition of Si and Asp compared with the salt-stressed celery (Figure 3).

Soluble sugar is the primary product of photosynthesis, and it plays a pivotal role as a building block of many indispensable macromolecules that regulate plant growth and development [71]. Also, sugar in plants is believed to be a candidate target of the osmoregulation system in response to salt stress [72]. Thus, it can be enriched when plants are subjected to salinity, in particular salt-tolerant genotypes [73]. Moreover, soluble sugar acts as a chelating agent, trapping the Na^+^ within starch granules for detoxification [73,74]. Furthermore, starch could play a critical role in detoxifying toxic ions by acting as a Na^+^-starch-binding granule [74]. In this study, the soluble sugar content in salt-stressed celery leaves was notably higher compared with that in CK (Figure 4A), which agrees with the findings by Gao [32] and Yin [73]. On the contrary, starch content strongly declined when NaCl was applied (Figure 4B), which is in line with the results in rice (both sensitive and tolerant genotypes against salt) [73,75,76]. Similarly, the salt stress resulted in reductions in both soluble protein and carotenoids, suggesting that the physiological status of celery was severely affected. Similar results of soluble protein were also reported in *Phaseolus vulgaris* [77] and *Lycopersicon esculentum* [78]. Several previous reports showed that the total carotenoids were increased, as affected by the salt stresses in many plants, due to the fact that the carotenoids played a protective role against oxidative damages [79,80]. Nevertheless, the carotenoids were clearly decreased in salt-stressed celery leaves in this study (Figure 4C), which is probably because of the photo-damage as a result of the loss of chlorophyll due to light absorbance. Our data are in line with the findings in wheat [81] and tomato [82]. Interestingly, the application of Si and Asp significantly reduced these detrimental influences caused by high salt content. Therefore, Si and Asp could synergistically alleviate the degree of salt stress in terms of nutritional status.

The external solution containing a high salt concentration inevitably instigated the ion imbalance or disturbances of ion homeostasis [1,4]. It has been established that there existed a competition between Na^+^ and K^+^, and thereby, the internal K^+^ declined under a high external NaCl environment, causing the K^+^ deficiency and, eventually, plant growth inhibition and ionic toxicity [83,84]. Indeed, solely NaCl-treated celery plants herein promoted the enhancement of Na^+^ while decreasing the internal K^+^ (Figure 5A,B). And we clearly detected a negative correlation (r = −0.83) between the Na^+^ and K^+^ (Figure 5E). Similar to K^+^, salt-stressed celery also showed lower tissue retention of Ca^2+^ and Mg^2+^ (Figure 5C,D), suggesting that Na^+^ was still negatively correlated with Ca^2+^ and Mg^2+^. This finding was further evidenced by Figure 5E, and the coefficient between Na^+^ and Ca^2+^ and Na^+^ and Mg^2+^ was −0.84 and −0.77, respectively. However, the addition of Si and Asp significantly ameliorated the reductions in K^+^, Ca^2+^, and Mg^2+^ (Figure 5). The Si-related alleviation of ionic toxicity during salt stress has been widely reported: the mechanism beyond it is that Si can restrict the uptake and transportation of Na^+^ and even mediate the compartmentalization of Na^+^ [85]. On the other hand, pioneering researchers found that exogenous amino acids could incite the stomatal opening and further modulate the ion’s transportation across the membrane, thereby improving salt tolerance [86]. Accordingly, the beneficial impacts of Si and Asp on the reduction in the ionic toxicity degree by salinity have been confirmed again.

It has been well-established that the addition of Si could regulate the defense system against oxidative stresses [37,38,41,60]. The stimulation of antioxidant enzyme activities by the supplementation of Si was frequently observed, rendering a protective role against oxidative damage on the cell membrane [40,41,46,47]. In this work, exogenous Si on salt-treated celery plants dramatically improved the concentration of SOD, POD, CAT, and APX (Figure 6), and we noticed that ROS accumulations (O_2_^·−^ and H_2_O_2_) declined accordingly (Figure 7). It can, therefore, be concluded that the salt toxicity that induced excess oxidative injuries was mitigated by the supplementation of Si. The data regarding the triggered antioxidant machinery of this study are in line with numerous previous studies [41,87]. Meanwhile, the leaf spraying of amino acids like Asp and proline improved the antioxidant enzyme activities under abiotic stresses, in accordance with the literature [18,32,83]. The amino acid per se is one of the important components of an antioxidant system in higher plants, and its actions involve not only the scavenging of free radicals but also osmoprotection and stress response [88,89]. Our results regarding the spraying of Asp and its influences on antioxidant enzymes and ROS content support these research findings (Figure 6 and Figure 7). As expected, the effect of the co-application of Si and Asp on the antioxidant defense system was the most pronounced. Succinctly, Si and Asp could reinforce antioxidant enzyme activities and concomitantly decrease antioxidative damages, especially in salt-stressed celery.

## 5. Conclusions

To sum up, this study first showed that 100 mM of NaCl-spiked celery “Si Ji Xiao Xiang Qin” developed salt toxicity. This phenomenon mainly included the following: seriously decreased plant growth parameters (whole plant biomass, shoot length, stem diameter, leaf length and width, and tap root length), declined photosynthetic capacity (*P*n, *T*r, *g*_s_, and chlorophylls), disturbed nutritional status (soluble sugar, starch, soluble protein, and carotenoids), interrupted ion uptake (Na, K, Ca, and Mg), and a diminished antioxidant defense system (antioxidant enzymes and ROS).

Conversely, the supplementation of Si and Asp, regardless of Si, Asp, or both, significantly reduced the degree of salt stress. Si and Asp may attribute this mitigation potential to the ameliorated parameters mentioned above.

Overall, exogenously applied Si and Asp could be an effective fertilization strategy in the alleviation of salt stress for celery cultivation.

## Figures and Tables

**Table 1:** The whole dry weight, leaf length, and stem diameter of celery as affected by 5 treatments.

Treatment	Whole Dry Weight (mg)	Leaf Length (cm)	Stem Diameter (mm)
CK	50.1 b	2.00 b	0.23 bc
NaCl	28.0 c	1.63 c	0.12 d
NaCl + Si	52.7 b	2.07 b	0.23 b
NaCl + Asp	49.2 b	2.02 b	0.20 bc
NaCl + Si + Asp	86.0 a	2.35 a	0.32 a

The displayed data are the means ± SE by *n* = 6 replicates. The different accompanied lowercase letters indicate the significant differences according to Duncan’s multiple comparison range test (One-way ANOVA) at *p* ≤ 0.05.

## Data Availability

Data sharing is not applicable to this article.
